# Genome-Wide Transcriptional Dynamics in the Companion Bacterial Symbionts of the Glassy-Winged Sharpshooter (Cicadellidae: *Homalodisca vitripennis*) Reveal Differential Gene Expression in Bacteria Occupying Multiple Host Organs

**DOI:** 10.1534/g3.117.044255

**Published:** 2017-07-13

**Authors:** Gordon M. Bennett, Rebecca A. Chong

**Affiliations:** * Department of Plant and Environmental Protection, University of Hawaii at Manoa, Honolulu, Hawaii 96822; † Department of Integrative Biology, University of Texas, Austin, Texas 78712

**Keywords:** bacteriomes, mutualism, co-evolution, genomics, obligate symbioses

## Abstract

The agricultural pest known as the glassy-winged sharpshooter (GWSS) or *Homalodisca vitripennis* (Hemiptera: Cicadellidae) harbors two bacterial symbionts, “*Candidatus* Sulcia muelleri” and “*Ca*. Baumannia cicadellinicola,” which provide the 10 essential amino acids (EAAs) that are limited in the host plant-sap diet. Although they differ in origin and symbiotic age, both bacteria have experienced extensive genome degradation resulting from their ancient restriction to specialized host organs (bacteriomes) that provide cellular support and ensure vertical transmission. GWSS bacteriomes are of different origins and distinctly colored red and yellow. While *Sulcia* occupies the yellow bacteriome, *Baumannia* inhabits both. Aside from genomic predictions, little is currently known about the cellular functions of these bacterial symbionts, particularly whether *Baumannia* in different bacteriomes perform different roles in the symbiosis. To address these questions, we conducted a replicated, strand-specific RNA-seq experiment to assay global gene expression patterns in *Sulcia* and *Baumannia*. Despite differences in genomic capabilities, the symbionts exhibit similar profiles of their most highly expressed genes, including those involved in nutrition synthesis and protein stability (chaperonins *dnaK* and *groESL*) that likely aid impaired proteins. *Baumannia* populations in separate bacteriomes differentially express genes enriched in essential nutrient synthesis, including EAAs (histidine and methionine) and B vitamins (biotin and thiamine). Patterns of differential gene expression further reveal complexity in methionine synthesis. *Baumannia*’s capability to differentially express genes is unusual, as ancient symbionts lose the capability to independently regulate transcription. Combined with previous microscopy, our results suggest that the GWSS may rely on distinct *Baumannia* populations for essential nutrition and vertical transmission.

Many insect groups have obligate associations with heritable microbes that enable novel ecological adaptations (Buchner 1965; Baumann 2005; Douglas 2015). In particular, plant sap-feeding insects (Order: Hemiptera) have ancient associations with bacteria that supplement their nutritionally imbalanced diets with the 10 essential amino acids (EAAs) and vitamins (McCutcheon and Moran 2010; Bennett and Moran 2015; Sudakaran *et al.* 2017). These bacteria are generally transmitted vertically and they are intracellularly restricted to specialized host organs called bacteriomes (Buchner 1965). Distinct bacteriome organs typically contain a single bacterial species even when a host relies on more than one complimentary symbiont as is common in the Auchenorrhyncha (*e.g.*, leafhoppers, cicadas, spittlebugs) (Buchner 1965; Braendle *et al.* 2003; Koga *et al.* 2013). However, in some host groups that have acquired novel symbionts more recently, such as sharpshooter leafhoppers (Cicadellidae: Cicadellinae), bacteria consistently occupy multiple bacteriomes (Buchner 1965; Moran *et al.* 2003, 2005b). As a result of ancient intracellular associations with host bacteriomes, tightly controlled vertical transmission, and small population sizes, symbiotic bacteria experience dramatic gene loss and rapid rates of molecular evolution (Moran 1996; Andersson and Kurland 1998; Wernegreen 2015). The impact of these forces on basic bacterial cell function remains poorly understood, particularly in complex symbiotic systems where hosts rely on multiple partners with different origins, levels of genome decay, and tissue associations.

To understand (a) how companion symbiont gene expression patterns are affected by the symbiotic condition, and (b) whether symbionts infecting multiple tissues exhibit distinct cellular functions, we conducted replicated transcriptomic sequencing (RNA-seq) of bacteriomes from the glassy-winged sharpshooter leafhopper (GWSS), *Homalodisca vitripennis*. GWSS relies on a complex symbiosis with two obligate bacteria for synthesis of the 10 EAAs (Wu *et al.* 2006; McCutcheon *et al.* 2007). Like many other hosts in the Auchenorrhyncha, GWSS retains the ancestral symbiont “*Candidatus* Sulcia muelleri” (*Bacteroidetes*; hereafter *Sulcia*), that has allied with the suborder since it emerged >280 MYA (Moran *et al.* 2003, 2005b; Bennett and Moran 2013). *Sulcia* is perhaps one of the oldest known symbionts; it has a small genome in GWSS (243 kb) and is responsible for the synthesis of eight EAAs (Moran *et al.* 2005b; Wu *et al.* 2006; McCutcheon *et al.* 2009a). In sharpshooters, the remaining EAAs are provided by “*Ca*. Baumannia cicadellinicola” (*Gammaproteobacteria*; hereafter *Baumannia*; Wu *et al.* 2006). *Baumannia* is derived from the replacement of the more ancient leafhopper symbiont “*Ca*. Nasuia deltocephalinicola” (*Betaproteobacteria*) in a common ancestor of sharpshooters >80 MYA (Moran *et al.* 2003; Bennett and Moran 2013). In GWSS, *Baumannia* retains a relatively large genome for an insect intracellular symbiont (686 kb) (Wu *et al.* 2006; Moran and Bennett 2014). It encodes many cellular capabilities lost in other ancient symbionts, including the potential to selectively regulate gene expression.

The acquisition of *Baumannia* coincided with the evolution of a novel bacteriome organ, which has also been found to occur in recent symbiont replacements in spittlebugs (Cercopoidea; Moran *et al.* 2003; Bennett and Moran 2013; Koga *et al.* 2013). Sharpshooter bacteriomes generally comprise paired red and yellow tissues located along the lateral edges of the abdomen (Figure 1, A and B) (Moran *et al.* 2005b). While the red bacteriome houses only *Baumannia*, the yellow one contains *Sulcia* and *Baumannia* (Buchner 1965; Kaiser 1980; Moran *et al.* 2003, 2005b). *Baumannia*’s presence in multiple host tissues, and its potential metabolic and functional flexibility, raises the intriguing possibility that it may have multiple roles in the symbiosis.

**Figure 1:**
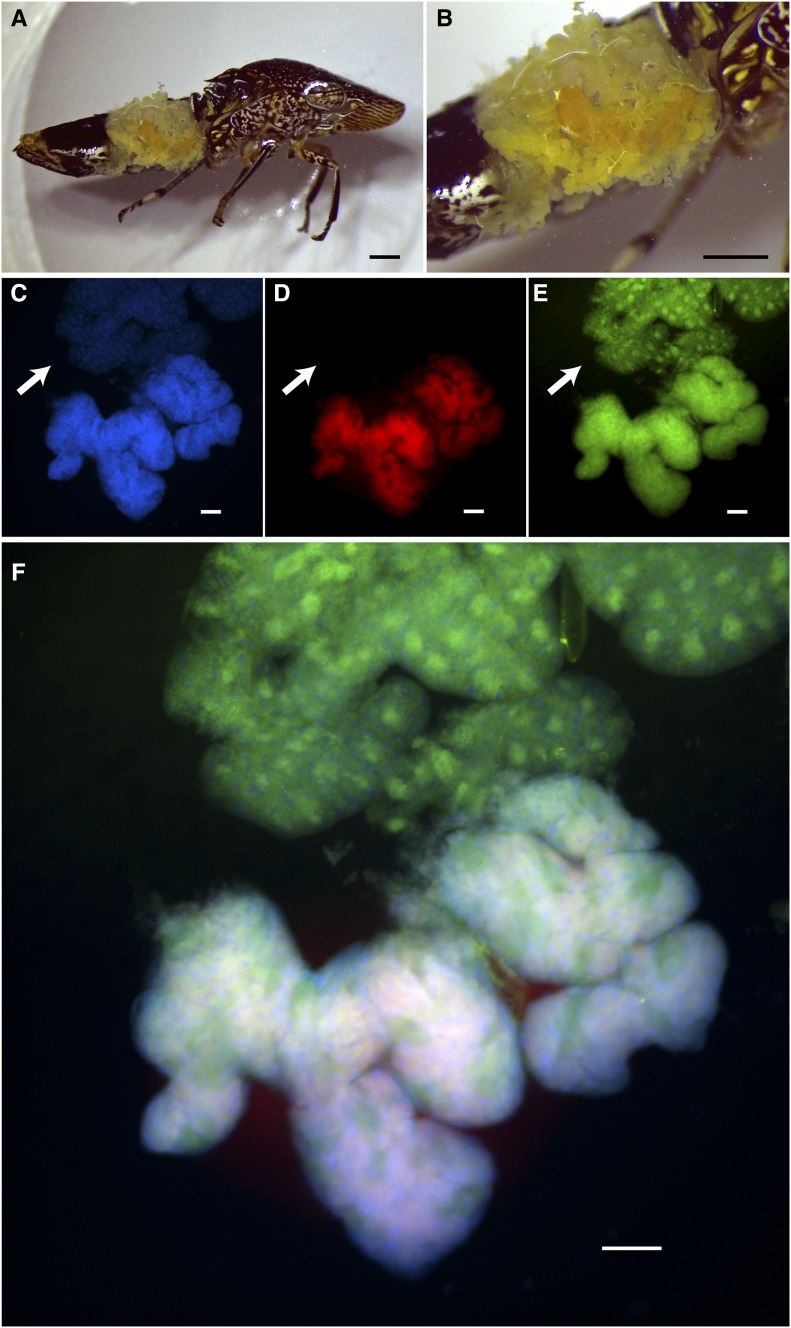
Bacteriome organs of the glassy-winged sharpshooter (GWSS), *Homalodisca vitripennis*. Microscopy of a female GWSS (A and B) dissected to show whole bacteriome structures. Black scale bars indicate 1 mm. (C–F) Fluorescence *in situ* hybridization (FISH) results for the red bacteriome (tissue in upper half) and yellow bacteriome (tissue in lower half). Images are split by fluorescence channel to show (C) DNA counterstained in blue (*i.e.*, host and bacterial DNA), (D) *Sulcia* in red, (E) *Baumannia* in green, and (F) a merged image of all three (C–E). Arrows in (D and E) show the red bacteriome tissues that exclusively contain *Baumannia*. White scale bar in (C–F) represents 100 μm.

One approach to better understand symbiont function is to profile their total gene expression patterns in the tissues that they occupy. These data can provide functional insight into dependent microbial partners that cannot be cultured or easily separated. For example, gene expression studies in the Aphid–*Buchnera* model system and a handful of other insect symbioses have shown that bacteria generally highly express genes involved in essential nutrition and protein homeostasis (*e.g.*, GroESL) (Fukatsu and Ishikawa 1993; Aksoy 1995; Charles *et al.* 1997; Wilcox *et al.* 2003; McCutcheon *et al.* 2009a; Stoll *et al.* 2009a). Microarray studies in *Buchnera* have further demonstrated that owing to loss of regulatory capabilities, gene expression is relatively static under conditions that should favor strong up-regulation of stress response and nutrition synthesis genes (Wilcox *et al.* 2003; Moran *et al.* 2005a; Stoll *et al.* 2009b). Here, our results show that symbioses between GWSS, *Sulcia*, and *Baumannia* also show typical high gene expression of chaperonin and nutrition genes despite differences in age and origin, underscoring the shared impact of the symbiotic condition. Intriguingly, *Baumannia* is capable of differentially expressing genes between bacteriomes, which includes those involved in essential nutrition synthesis. Combined with previous microscopy studies (Buchner 1965; Kaiser 1980), these results suggest that the host may rely on separate *Baumannia* populations for distinct symbiotic functions.

## Materials and Methods

### Fluorescence in situ hybridization microscopy

GWSS specimens were collected from crape myrtle (*Lagerstroemia indica*) at the University of Texas at Austin (UTA) in August of 2016 for fluorescence *in situ* hybridization (FISH) symbiont localization experiments. FISH analyses were conducted on whole bacteriomes of three replicate individuals following previous approaches with GWSS that determined *Baumannia* and *Sulcia* cell morphology (Moran *et al.* 2005b). Insect samples were initially fixed overnight in Carnoy’s solution. In order to eliminate potential autofluorescence, tissues were bleached in 6% H_2_0_2_–80% EtOH for 2 wk (Koga *et al.* 2009). Following bleaching, bacteriomes were rinsed with 100% EtOH and PBSTx, and hybridized overnight with the fluorescent probes, CFB319–rhodamine and PRO319–Alexa488, which target *Sulcia* and *Baumannia*, respectively (Moran *et al.* 2005b). DNA was counterstained with DAPI. Tissues were slide-mounted and visualized on a Nikon Eclipse TE2000-U epifluorescence microscope.

### Sample preparation and RNA sequencing

Three insect replicate pools for dissection and RNA sequencing were collected separately from *L. indica* on the UTA campus between June and August 2015. Insects were kept on *L. indica* for 48 hr in controlled growth conditions (25°, 12 hr light/dark cycle) to mitigate potential environmental effects on gene expression. For each replicate, 14 mature females were CO_2_-treated and whole bacteriomes immediately dissected in Buffer A (25 mM KCl, 35 mM Tris, 100 mM EDTA, and 250 mM sucrose) and preserved in RNAlater (Ambion). Tissue pools were then centrifuged at 4° for 15 min at 13,000 × *g* and RNAlater decanted. RNA was extracted with TRIzol (Invitrogen) and residual DNA removed with DNA-*free* (Ambion). Total RNA was sent to the UTA Genomic Sequencing and Analysis Facility for sequencing. Ribosomal RNA was depleted using a Ribo-zero Magnetic Gold Epidemiology kit (Epicentre). Strand-specific libraries were prepared with a deoxy-UTP protocol and sequenced on an Illumina HiSeq 2500 (20–25 million, 2 × 125 bp paired-end reads for a mean insert size of 180 bp). Reads were demultiplexed, adapter trimmed, and quality filtered with Trimmomatic v0.32 (program settings: ILLUMINACLIP:2:20:10:1 LEADING:20 TRAILING:20 SLIDINGWINDOW:4:20 MINLEN:50) (Bolger *et al.* 2014).

### Transcriptome and genome mapping verification

Expression counts for downstream analyses were obtained by read mapping to the previously sequenced genomes for *Baumannia* (NCBI# CP000238) and *Sulcia* (NCBI# CP000770) (Wu *et al.* 2006). Bacterial reads were extracted by mapping with Bowtie2 v2.2.6, and residual rRNA-mapping reads were excluded *in silico* (Langmead and Salzberg 2012). To assess whether the different bacteriome types harbor genetically distinct *Baumannia* populations, we estimated genome-wide nucleotide divergence for all protein-coding genes between bacteriome transcript libraries. We also performed this analysis to assess potential population-level divergence between Texas populations (collected in 2015) used in this study and the sequenced reference genomes from California (collected in 2004; Wu *et al.* 2006). Both scenarios could lead to biased read mapping in rapidly diverging genes. *Sulcia* was not included in these analyses since it occupies only the yellow bacteriome and has an exceptionally low rate of molecular evolution (Bennett *et al.* 2014, 2016). Red and yellow bacteriome read libraries were *de novo* assembled from Replicate 1 with Trinity v2.1.1 (Table 1) (Grabherr *et al.* 2011). *Baumannia* transcripts were identified and filtered with NCBI-BLASTN v2.2.30+ searches against the annotated *Baumannia* genome (program settings: -max_target 5, -eval 0.01, -best_hit_overhang 0.25, -best_hit_score_edge 0.05). Transcripts were annotated with Glimmer v3 and assembled to the sequenced genome (Delcher *et al.* 2007). All identified protein-coding genes were extracted and pairwise aligned with MAFFT L-INS-I; percentage divergence was estimated in Geneious v9.1 (Kearse *et al.* 2012; Katoh and Standley 2013).

**Table 1:** Replicated RNA sequencing and bacterial symbiont mapping results for *Homalodisca vitripennis* bacteriomes

Replicate	Bacteriome	Reads	Symbiont	Mapped	% of Reads
Rep 1	Red	24,733,585	*Baumannia*	19,783,309	80.33
			*Sulcia*	26,062	0.13
	Yellow	20,763,561	*Baumannia*	2,875,395	15.00
			*Sulcia*	9,820,142	47.51
Rep 2	Red	23,033,014	*Baumannia*	18,586,145	83.94
			*Sulcia*	17,156	0.12
	Yellow	20,787,994	*Baumannia*	2,876,250	15.56
			*Sulcia*	9,277,730	47.88
Rep 3	Red	22,935,642	*Baumannia*	16,197,931	72.40
			*Sulcia*	53,567	0.44
	Yellow	20,559,249	*Baumannia*	3,283,895	16.62
			*Sulcia*	8,675,965	44.31

### Strand-specific gene expression across symbiont genomes

Since libraries were prepared with a strand-specific protocol, we undertook two strategies to examine and control for antisense RNA expression. Both antisense RNA (asRNA) expression and transcriptional bleed-through from coding sequences on the opposite strand can inflate expression estimates for protein-coding genes (Zhao *et al.* 2015). Thus, we first predicted noncoding RNAs (ncRNAs) and asRNAs with Rockhopper v100 for *Sulcia* and *Baumannia* (both red and yellow libraries were used in the latter case; program settings: mismatches = 0.15, seed length = 0.33, and minimum expression = 0.5) (McClure *et al.* 2013). Predicted RNAs were annotated and included in mapping files for expression analyses (see *Results and Discussion*). Second, sequence alignment map (SAM) files from Bowtie2 mapping were split into strand-specific read pools with Picard Tools v2.2.2 (Broad Institute: http://broadinstitute.github.io/picard/). Strand-specific reads were then used to estimate expression values for predicted genes in their sense and antisense orientation (*i.e.*, within predicted gene frames on both strands) with EDGE-pro v1.3.1 (program settings: -w 100, -i 40, -n 0, partial counting). EDGE-pro is designed specifically for bacterial genomes that exhibit high gene density and overlapping coding regions (Magoc *et al.* 2013). Gene expression values were estimated as transcripts per million (TPM) from sense read counts (Wagner *et al.* 2012). Gene functions for downstream discussion and analysis were verified with the EcoCyc database (Keseler *et al.* 2011).

### Baumannia differential gene expression between bacteriomes

Genome-wide differential gene expression was estimated for sense gene predictions (*i.e.*, protein-coding sequences and predicted RNAs) between *Baumannia* populations from the red and yellow bacteriomes. Raw read counts for each replicate from EDGE-pro were exported for differential expression analyses with DEseq2 in the R Bioconductor package (Love *et al.* 2014; Huber *et al.* 2015). Counts were normalized and log_2_ fold-change values were estimated for each gene. Statistical *P*-values were adjusted with Benjamini–Hochberg (BH) correction for multiple comparisons with a false-discovery rate of *P* ≤ 0.05. We further tested significant differential expression of genes at multiple log_2_ fold change thresholds (FCT) of 0.0, 0.5, and 1.0 (>2× fold expression change) to help distinguish significant gene enrichments from random variants and false positives (program settings: lfcThreshold = *n*, altHypothesis = greaterAbs) (Love *et al.* 2014). Since initial analyses revealed striking differences in *Baumannia* read depth between bacteriomes, we further performed these analyses on a dataset with equalized read depth (Table 1). For these datasets, the red bacteriome libraries were down sampled to 3 million reads to verify robustness of our results. Finally, differentially expressed protein-coding genes for each FCT were binned into Clusters of Orthologous Genes (COGs) (Tatusov *et al.* 2003). Fisher’s exact test was used to test for significant COG enrichments with a BH correction in R.

### Data availability

Symbiont mapping reads for each replicate were submitted to the GenBank Single Read Archive (SRA) database under accession numbers SRR241705–SRR241717.

## Results and Discussion

### Localization and population divergence in GWSS symbionts

To verify the tissue-specific associations of *Baumannia* and *Sulcia* in GWSS, FISH microscopy was performed on red and yellow bacteriomes (Figure 1). Nonfluorescence microscopy illustrates the red and yellow bacteriome tissues in the GWSS abdomen (Figure 1, A and B). FISH confirms that the red bacteriome exclusively harbors *Baumannia*, while the yellow contains both *Baumannia* and *Sulcia* (Figure 1, C–F). We note that autofluorescence was not observed in bleached tissues at the wavelengths of the selected fluorescent probes. *Baumannia* appears to be integrated throughout the yellow organ. It may co-occupy bacteriocytes with *Sulcia*, as has been observed previously in two other sharpshooter species, *Graphocephala coccinea* and *Cicadella viridis* (Buchner 1965; Kaiser 1980). Sequence divergence estimates further show that *Baumannia* is highly conserved between Texas and California populations, and also between bacteriome types (99.9% average sequence identity for all protein coding genes [*n* = 605]; Supplemental Material, Table S1). The latter finding supports microscopy observations that *Baumannia* cells in the red and yellow bacteriomes are derived from a single inoculum that is sorted into the two bacteriomes during early embryonic development (Buchner 1965; Kaiser 1980).

### Genome-wide gene expression in Sulcia and Baumannia

To investigate genome-wide gene expression patterns in *Sulcia* and *Baumannia* in GWSS, we sequenced complete bacterial transcriptomes from three replicate dissections of the red and yellow bacteriomes. Sequencing yielded 20–25 million quality-filtered reads for each library (Table 1). Read mappings to bacterial genomes confirmed that *Sulcia* does not infect the red bacteriome and that *Baumannia* is present in both (Figure S1 and Table 1). asRNA and ncRNA predictions identified 18 genes in *Baumannia* (avg. length = 333 bp [52–1047 bp]) and none for *Sulcia* (Table S2). Despite asRNA detection efforts, we still observed genome-wide antisense expression in both symbionts (Figure S1 and Table S2). Approximately 9% of *Sulcia* mapping reads are expressed in the antisense orientation. In *Baumannia*, 4 and 7% of reads map to the antisense strand in the yellow and red bacteriomes, respectively. The difference between bacteriomes is partly explained by the finding that *Baumannia* more highly expresses several asRNAs in the red bacteriomes (see below). Finally, replicates show high correspondence in both symbionts (discussed below), particularly regarding the differential expression of select functions. Since our study was done in triplicate on samples that were obtained and sequenced separately, a reverse transcription quantitative PCR validation (RT-qPCR) step was not undertaken. Many recent studies have demonstrated correlation between genome-wide sequencing approaches and RT-qPCR (Nagalakshmi *et al.* 2008; Wang *et al.* 2009; Camarena *et al.* 2010; Raghavan *et al.* 2012; Wu *et al.* 2014; Pombo *et al.* 2017).

In general, the ratio of sense *vs.* antisense read counts for each gene was above one (*i.e.*, higher on the sense strand, as would be expected for valid gene predictions; Table S2). However, exceptions were observed in *Baumannia* (*n* = 35 genes) and *Sulcia* (*n* = 32 genes). Several of these genes include predicted RNAs (tRNAs and ncRNAs), suggesting bistranded expression or incorrect gene orientation prediction. For protein-coding genes, nearly all are neighbor to genes on the opposite strand that exhibit higher read counts, often by an order of magnitude (∼90% in both symbionts). For example, the *Sulcia iscS* gene shows higher expression on the antisense strand (22,897 *vs.* 6596 reads), but is flanked by the highly expressed *trpE* gene on the opposite strand (68,711 reads). These results suggest that estimation of gene expression in symbiotic bacteria may be confounded by high antisense transcription. In order to properly estimate gene expression, strand-specific sequencing approaches should be undertaken in these systems.

**Figure 2:**
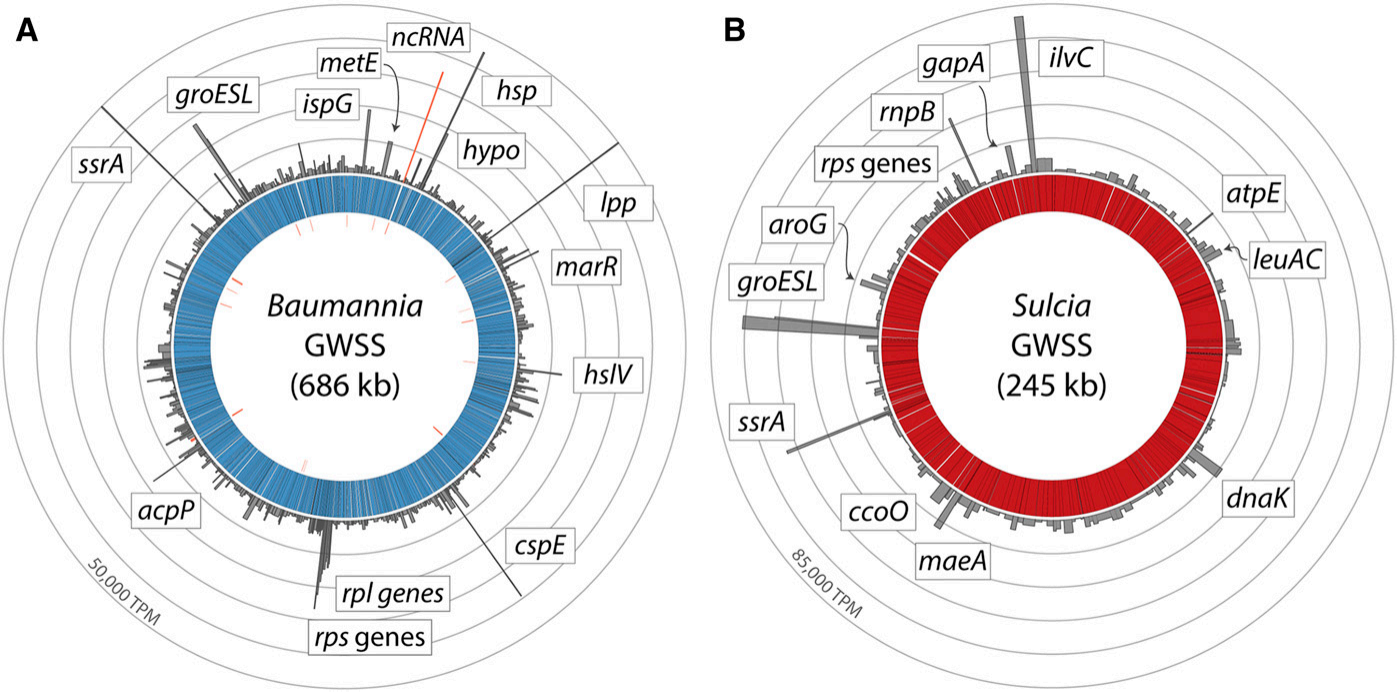
Genome-wide gene expression profiles for (A) *Baumannia* and (B) *Sulcia* symbionts from the sharpshooter insect host, *Homalodisca vitripennis* (GWSS). Bar graphs show per gene TPM estimates for replicate 1 (see Table 1) of the yellow bacteriome. Inner rings and striations show the bacterial genomes and individual genes. Tick marks on the innermost ring show the locations of predicted noncoding RNAs. A selected set of the most highly expressed genes is labeled with gene shorthand names; not all genes discussed are labeled owing to space constraints. See Supplemental Material for complete TPM values (Table S1) and stranded expression counts (Figure S1).

In *Sulcia*, the global gene expression profiles do not differ significantly between *Sulcia* replicates (*P* = 0.754, Kruskal–Wallis rank sum test). The top 30 most highly expressed genes (∼90th percentile) are predominately involved in EAA synthesis, buffering degraded protein function, and other basic cellular processes (see Figure 2 and Table S2 for TPM values). Specific genes include protein chaperonins (*groESL* and *dnaK*), EAA metabolite synthesis (isoleucine and valine [*ilvBC*], leucine [*leuAC*], phenylalanine and tryptophan [*aroEG* and *trpBF*], threonine [*thrB*], and lysine and methionine [*asd* and *dapD*]), transcription and translation (*tufA*, *rnpB*, *rpmA*, *rplA*, and *rpsDEKMNU*), pyruvate synthesis (*maeA*), transcription release factor (prfA), and energy synthesis (*gapA*, *ccoN*, and *atpE*). Finally, one of the most highly expressed genes is an *ssrA* RNA that rescues stalled ribosome function from damaged mRNA. This gene may participate in aiding transcriptional machinery and protein production impaired by high mutation rates or low transcriptional fidelity.

In *Baumannia*, the top 30 most highly expressed genes (∼95th percentile) reflect its larger functional content and nutritional role of providing two EAAs and B vitamins (see Figure 2 and Table S2 for TPM values). In both the red and yellow bacteriomes, the most highly expressed *Baumannia* genes include an *ssrA* gene, protein chaperonins and thermal shock response genes (*cspE*, *hsp*, and *groESL*), and methionine synthase (*metE*) that is involved in the final catabolic step of methionine production (Figge 2006). One of the most highly expressed genes is a predicted ncRNA (360 bp in length). Subsequent searches against the RFAM database identified this gene as a potential Rnase P, which cleaves precursor sequences from tRNA molecules (Nawrocki *et al.* 2015). Otherwise, the roles of ncRNAs and asRNAs are unclear, but these genes may be involved in the regulation of gene expression (Repoila and Darfeuille 2009). Excluding genes that are differentially expressed, the other most highly expressed *Baumannia* genes are involved in cell membrane synthesis (*acpP* and *lpp*), DNA metabolism and protection (*ssb*), isoprenoid catabolism (*ispG*), transcription and translation (*rpsCJOQS* and *rplBCDPVW*), and protein and mRNA decay (*hslV* and *orn*, respectively). In the red bacteriome, several nutrition synthesis genes emerge among the 95th percentile because they are highly differentially expressed between bacteriomes. These include genes involved in the synthesis of biotin (*bioBF*), thiamine (*thiC*), and histidine (*hisCG*) (discussed below).

Despite the differences in origin and genomic capabilities of their symbiotic associations with GWSS, *Sulcia* and *Baumannia* exhibit important similarities in their gene expression profiles. Both highly express a suite of genes involved in the synthesis of essential nutrition required by each partner, underlying their central role in the symbiosis. This is congruent with genome-wide protein-level gene expression estimates of *Sulcia* and its coprimary symbiont *Hodgkinia* in a cicada host (Cercopoidea; McCutcheon *et al.* 2009a,b). They also highly express genes involved in translation and other basic cellular metabolisms, which are among the most highly expressed functional categories in free-living bacteria, including *Escherichia coli* (Karlin *et al.* 2001). *Sulcia* and *Baumannia* gene expression profiles further highlight the endemic challenge symbiotic bacteria experience – degraded protein function (McCutcheon and Moran 2012). One apparent coping mechanism is the constitutive overexpression of protein chaperonins and heat shock proteins by symbionts. Chaperonins in free-living bacteria buffer enzyme function against the effects of environmental extremes, oxidative stress, and genetic drift, which are major forces shaping symbiont genome evolution (Williams *et al.* 2010; McCutcheon and Moran 2012; Sabater-Muñoz *et al.* 2015). In bacterial symbionts, chaperonins likely aid in the proper folding of proteins debilitated by the accumulation of deleterious mutations (Moran 1996; Fares *et al.* 2004; Kupper *et al.* 2014). The overexpression of chaperonins has been observed in a range of insect symbioses, including *Sulcia* and *Hodgkinia* in cicadas, *Buchnera* in aphids, *Blattabacterium cuenoti* in cockroaches, *Wigglesworthia glossinidia* in tsetse flies, “*Ca*. Blochmannia floridanus” in carpenter ants, and *Sodalis* sp. in weevils (Fukatsu and Ishikawa 1993; Aksoy 1995; Charles *et al.* 1997; McCutcheon *et al.* 2009a,b; Stoll *et al.* 2009b). The wide range of systems that have evolved fixed, high expression of GroEL points to its central role in stabilizing insect bacterial symbioses. Nevertheless, high constitutive expression of GroESL is likely costly to hosts. It is known to be highly expressed as protein in pea aphids, requiring extensive cellular resources (Baumann *et al.* 1996; Sabater-Muñoz *et al.* 2015). Furthermore, it may also impose ecological constraints on plant–insect interactions. It was recently found that high levels of *Buchnera* GroEL in aphid saliva can trigger a plant immune response, lowering insect fecundity and possibly limiting host ecological range (Chaudhary *et al.* 2014).

### Select metabolic functions in Baumannia are differentially expressed between bacteriomes

**Figure 3:**
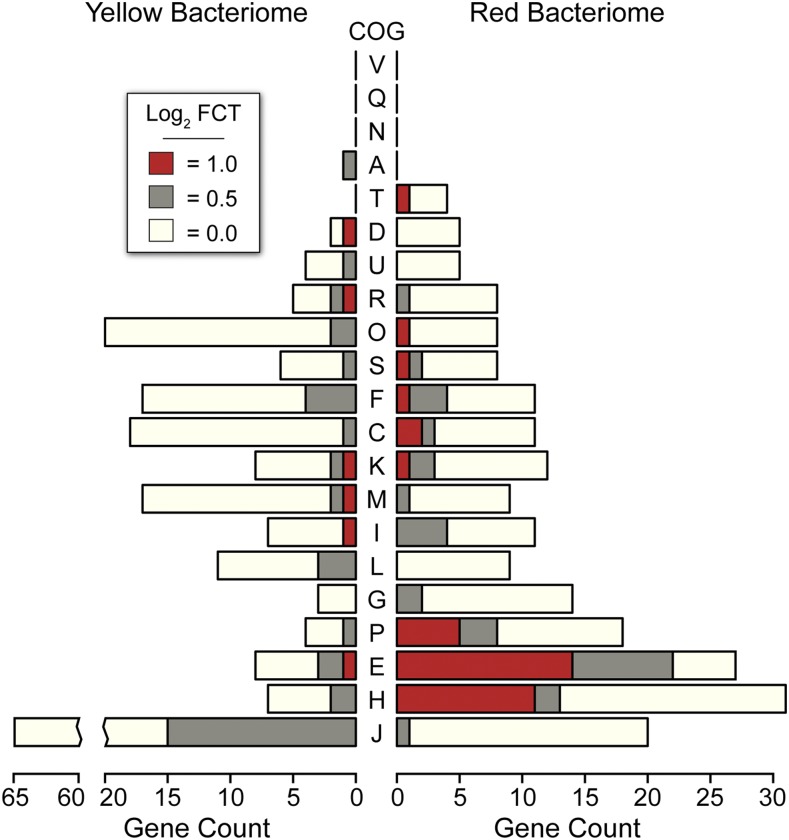
Clusters of orthologous genes (COG) enrichment for each fold change threshold (FCT) of differentially expressed *Baumannia* genes (see inset legend). COG categories are as follows: A, RNA processing and modification; B, chromatin structure and dynamics; C, energy production and conversion; D, cell cycle control and mitosis; E, amino acid metabolism and transport; F, nucleotide metabolism and transport; G, carbohydrate metabolism and transport; H, coenzyme metabolism; I, lipid metabolism; J, translation; K, transcription; L, replication and repair; M, cell wall/membrane/envelop biogenesis; N, cell motility; O, post-translational modification, protein turnover, and chaperone functions; P, inorganic ion transport and metabolism; Q, secondary structure; T, signal transduction; U, intracellular trafficking and secretion; Y, nuclear structure; Z, cytoskeleton; R, general functional prediction only; S, unknown (Tatusov *et al.* 2003).

**Figure 4:**
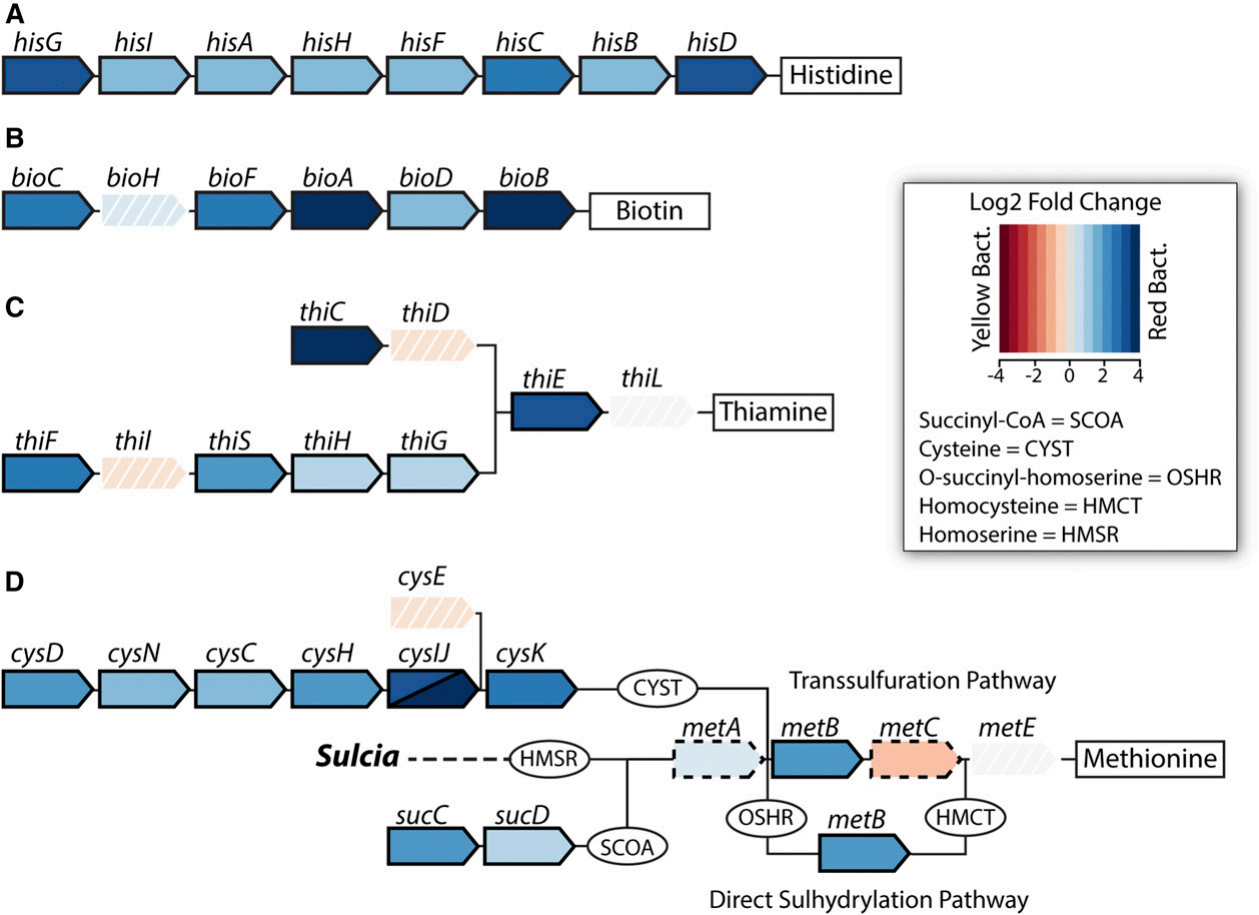
Differential gene expression values of *Baumannia* genes between bacteriome tissue types related to nutrition synthesis pathways: (A) histidine, (B) biotin, (C) thiamine, and (D) methionine (see Figure 1). Individual genes are represented by arrow-shaped polygons with gene names above. Intermediate metabolites are shown for some pathways and abbreviated (see legend). Genes with solid borders are significantly differentially expressed at a log_2_ fold change threshold (FCT) of 1.0 (>2× fold change). Genes with dashed borders show moderate significant differential expression at FCT = 0.5. Genes with white borders and hash marks are not significantly differentially expressed. Genes shown in shades of blue are more highly expressed in the red bacteriome, and genes shown in red are more highly expressed in the yellow bacteriome (see legend).

The occupation of multiple bacteriomes by an obligate heritable symbiont is unusual in insects, particularly in the Auchenorrhyncha (Buchner 1965; Koga *et al.* 2013). Thus, we hypothesized that given *Baumannia*’s expanded functional capacity, it may differentially express genes involved in distinct symbiotic activities between the bacteriomes that it occupies. To test this question, we conducted a replicated, tissue-specific differential expression RNA-seq experiment. To further verify the robustness of our sequencing effort, analyses were performed on total reads and datasets that down-sampled the red bacteriome libraries from >15 to 3 million reads in order to match sequencing depth in the yellow bacteriome. *Baumannia* indeed exhibits differential expression of genes between the red and yellow bacteriomes (Figure 3, Figure 4, Figure S2, and Figure S3). The number of differentially expressed genes varies slightly between analyses that account for differences in sequencing depth between bacteriomes. At an FCT = 1.0 (>2× expression difference), results using total reads had only two more differentially expressed genes, *aroH* and an ncRNA, for a total of 57 genes (discussed individually below; see Table S3). *Baumannia* sequencing replicates further demonstrate high correspondence in their differentially expressed genes (see Figure S3 for genome-wide expression variance). For simplicity, we categorically refer to significant differentially expressed genes at each FCT threshold of 0.0, 0.5, and 1.0 as showing low, moderate, and high differential expression. *Results and Discussion* are presented for total read analysis at FCT = 1 (>2 × expression difference), unless noted otherwise.

In the yellow bacteriome, differentially expressed genes are categorically enriched for translation (J) and post-translational modification (O) only under an FCT = 0.0 (*P* <0.001; Figure 3). A total of eight genes show high differential expression of >2× fold change (FCT = 1.0; Table S3). Protein-coding genes are involved in a broad range of functions that include cell envelope integrity (*lpp*), glycine cleavage (*gcvT*), aspartate catabolism (*aspA*), fatty acid metabolism (*accD*), tRNA modification (*gidA*), and methionine and oxidative stress transcription repressors (*metJ* and *marR*-like, respectively). Genes that are moderately up-expressed (*i.e.*, FCT = 0.5) also vary widely across metabolic and cellular activities, including amino acid metabolisms (*metC* and *folCD*), RNA processing and decay (*orn*), proteolysis and heat stress response (*clpP* and *ibpA*), translation (*rplBPVW*), and purine and pyrimidine synthesis (*purNM* and *pyrD*), among others (Table S3). The collective functional role of these highly differentially expressed *Baumannia* genes is unclear. Several genes appear to participate in incomplete genetic pathways. For example, *gcvT* is the sole retained gene of a four-enzyme complex involved in glycine cleavage, which is important in the synthesis of several cellular materials (*e.g.*, purines, cell wall, and protein synthesis; Okamura-Ikeda *et al.* 2003). Similarly, *aspA* has a predicted role in the TCA cycle, but this pathway is incomplete in *Baumannia* (Asano *et al.* 2005). Finally, the roles of differentially expressed transcription regulatory genes appear to be correlated with observed expression differences between bacteriomes. Although the role of the *marR*-like transcription repressor is unknown, *mar*-like genes are involved in the regulation of over 80 genes across a variety of cellular functions (Alekshun and Levy 1999; Barbosa and Levy 2000). The EAA methionine repressor gene, *metJ*, also shows higher expression correlated with the significant down-expression of the methionine synthesis gene, *metB*, that it is known to specifically regulate (Figge 2006).

In the red bacteriome, *Baumannia* highly differentially expresses 49 genes that are categorically enriched for amino acid synthesis (E; *P* <0.001) and vitamin and cofactor synthesis (H; *P* <0.001) regardless of FCT (Figure 3 and Figure 4; see also Table S3). Aside from nutrition synthesis, *Baumannia* highly expresses genes involved in a range of functions, including purine synthesis (*purBH*), redox reactions (*gnd* and *nadB*), and cell membrane synthesis (*dedA* and *pssA*). The genes also include two asRNAs, which have unknown functions. The rest of the genes that are more highly expressed in the red bacteriome are involved in nutrition synthesis. These genes include the entire histidine synthesis pathway (*hisABCDFGHI*), genes in the linked methionine and cysteine synthesis pathways (methionine [*metBF*], sulfur [*cysDNCHIJK*], transport [*cysUW*], transcription regulation [*cysB*], and succinyl-CoA [*sucCD*]), and several genes involved in the steps of aromatic EAA synthesis that *Baumannia* still partially encodes (*aroH* and *aspC*). An ABC amino acid transporter is also among the highly differentially expressed genes, and could play a part in increased amino acid exchange with the host. Genes involved in essential B vitamin synthesis are also among those more highly expressed in the red bacteriome, including biotin (*bioABCDF*), thiamine (*thiCEFGS*), and riboflavin (*ribB*). Several other genes that are moderately up-expressed (FCT = 0.5) complement these nutrition synthesis pathways, including methionine (initiating step, *metA*), cysteine transport (*fliY* and *yecC*), amino acid metabolism (aspartate [*aspC*], glycine [*glyA*]), and riboflavin (*ribA*) (Table S3).

The differential expression of nutrition synthesis genes implies that the host may rely on this *Baumannia* population primarily to balance the nutritional input required by each symbiotic partner. The differential expression of the complete histidine pathway, certain methionine and B vitamin synthesis genes, and related accessory and transport genes supports this hypothesis. For the synthesis of methionine, the differential expression of certain genes reveals complexity in this pathway, possibly owing to its reliance on metabolites from several other pathways (cysteine and succinyl-CoA) and flexible use of the transsulfuration (TS) or direct sulfhydrylation (DS) pathways (Hwang *et al.* 2002; Hacham *et al.* 2003). Both *Baumannia* populations appear to synthesize methionine, as *metE* is one of the most highly expressed genes in both bacteriomes (Figure 2). However, the intermediate synthesis enzyme, gamma-synthase (MetB), is more highly expressed in the red bacteriome (Figure 4). MetB is a flexible enzyme that is capable of skipping intermediate steps in methionine synthesis (*e.g.*, MetC conversion of cystathione to homocysteine in the TS pathway) to complete the DS pathway (Hacham *et al.* 2003; Figge 2006). Intriguingly, the *metC* locus shows moderate up-expression (FCT = 0.5) in the yellow bacteriome (Figure 4). The reciprocal expression patterns of these enzymes suggest that the red bacteriome may rely more on the DS pathway, which can bypass steps in the TS pathway, including MetC activity (Figge 2006). The *metC* enzyme degrades cysteine (Awano *et al.* 2003), which is required for the production of biotin, thiamine, and CoA (Guédon and Martin-Verstraete 2006). All of these synthesis pathways are highly up-expressed in the red bacteriome, and *metB* may offer a way to reduce potential metabolic conflict (Figure 4).

### Conclusion

It is unusual that *Baumannia* retains such flexibility to differentially express genes between host tissues and within certain functional categories. Symbionts generally lose transcriptional control mechanisms, and they demonstrate an attenuated ability to control transcript expression even when these are retained (Wilcox *et al.* 2003; Moran *et al.* 2005a; Stoll *et al.* 2009b; McCutcheon and Moran 2011). *Baumannia* still retains a suite of genes capable of regulating RNA synthesis. It still encodes nutrition-related transcription factors (TF) for methionine (*metJ* and *metR*), biotin (*birA*), and cysteine (*cysB*) that generally show expression levels congruent with differential expression patterns between bacteriomes. For example, *cysB* is highly up-expressed along with the cysteine and sulfur metabolism pathways in the red bacteriome, while *metJ* that negatively regulates *metB* is more highly expressed in the yellow bacteriome. Several other differentially expressed pathways, such as histidine, have lost their TFs and may rely on alternative regulatory mechanisms. Histidine regulation can rely on *relA* and ppGpp alarmone production, which is partially retained by *Baumannia* (Kulis-Horn *et al.* 2014). *Baumannia* also retains several sigma factors (*rpoD* and *rpoH*) that regulate expression of stress response genes and protein chaperonins. Finally, whether or not the differential expression of genes translates to the protein level in *Baumannia* is a question for future work. Post-transcriptional regulatory mechanisms may play a large part in gene expression control in this system (*e.g.*, ncRNAs) as observed in *Buchnera* from pea aphids (Hansen and Degnan 2014). Ultimate control of gene expression and nutrition synthesis may further lie with host-level regulatory and feedback mechanisms as was found for arginine (also in pea aphids) (Poliakov *et al.* 2011; Price *et al.* 2014).

In contrast to *Baumannia*, *Sulcia* lineages show conservation in gene expression patterns, even across divergent host systems. *Sulcia* from cicadas and leafhoppers diverged well over 100 MYA, yet they exhibit highly conserved genome-wide gene expression patterns, and also correlated protein and transcript expression levels (see McCutcheon *et al.* 2009a). These similarities include many of the same EAA genes (*e.g.*, *aroG*/*pheA*, *ilvC*, and *leuAC*) and the same set of chaperonins observed more widely across symbionts (*e.g.*, *groEL* and *dnaK*), among others. These patterns suggest that the mechanisms shaping gene expression in *Sulcia* may be highly conserved across the major Auchenorrhyncha host lineages. Furthermore, *Sulcia* protein and transcript expression levels also appear to be highly correlated. It is possible that *Sulcia* (or its hosts) are unable to modulate gene expression at these levels, and may rely on other regulatory mechanisms.

The reason *Baumannia* is maintained in two bacteriomes after millions of years, particularly in contrast to other Auchenorrhyncha symbioses (Buchner 1965; Koga *et al.* 2013), remains speculative. A plausible scenario is that GWSS relies on the two populations for distinct functions. The enriched expression of nutrition synthesis genes in the red bacteriome suggests that this population may have a central role in balancing total nutrition. By contrast, *Baumannia* in the yellow bacteriomes, which exhibits up-expression of a few genes with unclear functional correspondence, may be required for transovarial transmission. Early cytological work by Paul Buchner, H.J. Müller, and Barbara Kaiser provides some support for this hypothesis. They found that in several related sharpshooter species, *Baumannia* also coinfects red and yellow tissue homologs (Buchner 1965; Kaiser 1980). In the yellow bacteriomes, *Baumannia* and *Sulcia* colocalize to a region known as the “infection mound,” which buds off to ferry symbionts to developing bacteriome tissues. Symbionts, which are comixed within these cells, are subsequently sorted into bacteriome tissues during embryonic development. The red bacteriome appears to be a dead-end for that *Baumannia* population, as hosts do not transmit bacteria from these tissues to the ovariole. This observation could help to explain why bacteriome-specific populations do not show nucleotide sequence divergence. Given the relative youth of the red bacteriome, these tissues may not have evolved transmission mechanisms to passage symbionts. Thus, in order to ensure immediate and stable inheritance of both symbionts, *Baumannia* and its host may have co-opted the transmission mechanisms of the yellow bacteriome, which have faithfully transmitted *Sulcia* for 280 MY (Moran *et al.* 2005b).

## Supplementary Material

Supplemental material is available online at www.g3journal.org/lookup/suppl/doi:10.1534/g3.117.044255/-/DC1.
