# Corrigendum

**DOI:** 10.1534/g3.119.400095

**Published:** 2019-03-28

In the article by G. M. Bennett and R. A. Chong (*G3: Genes|Genomes|Genetics* 7: 3073-3082) entitled “Genome-Wide Transcriptional Dynamics in the Companion Bacterial Symbionts of the Glassy-Winged Sharpshooter (Cicadellidae: *Homalodisca vitripennis*) Reveal Differential Gene Expression in Bacteria Occupying Multiple Host Organs” on page 3076 in the Data Availability Statement, there was an error in the GenBank Single Read Archive (SRA) database accession numbers. The text has now been corrected to cite SRR4241705-SRR4241717.

