# Supplementary figures and images for: Genome-Wide Transcriptional Dynamics in the Companion Bacterial Symbionts of the Glassy-Winged Sharpshooter (Cicadellidae: Homalodisca vitripennis) Reveal Differential Gene Expression in Bacteria Occupying Multiple Host Organs
Source: G3 (Bethesda). 2017 Jul 13;7(9):3073–82. doi: 10.1534/g3.117.044255 (PMC5592932; doi:10.1534/g3.117.044255)

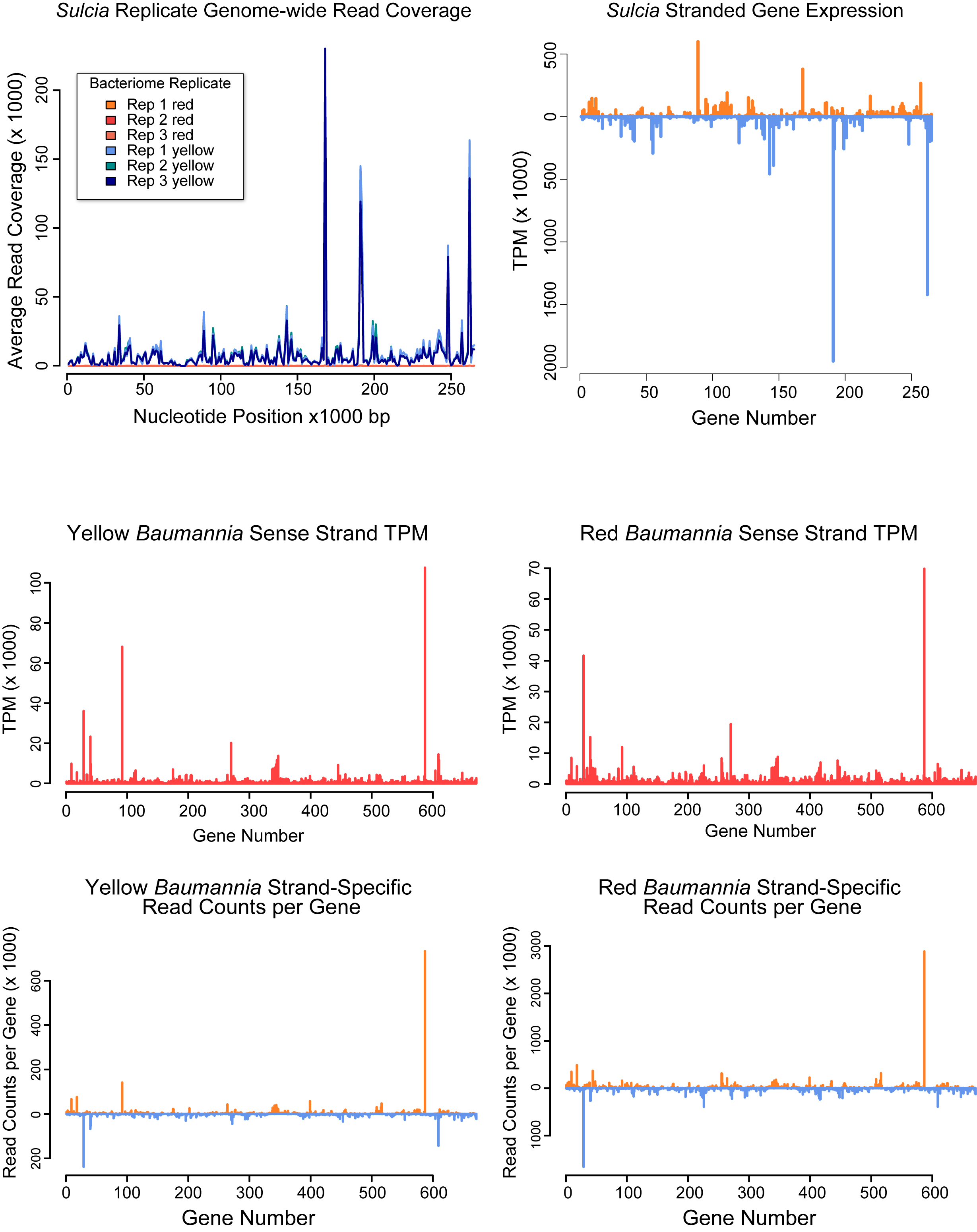

Supplement: Supplementary file 1 [file 3073FigureS1.tif]

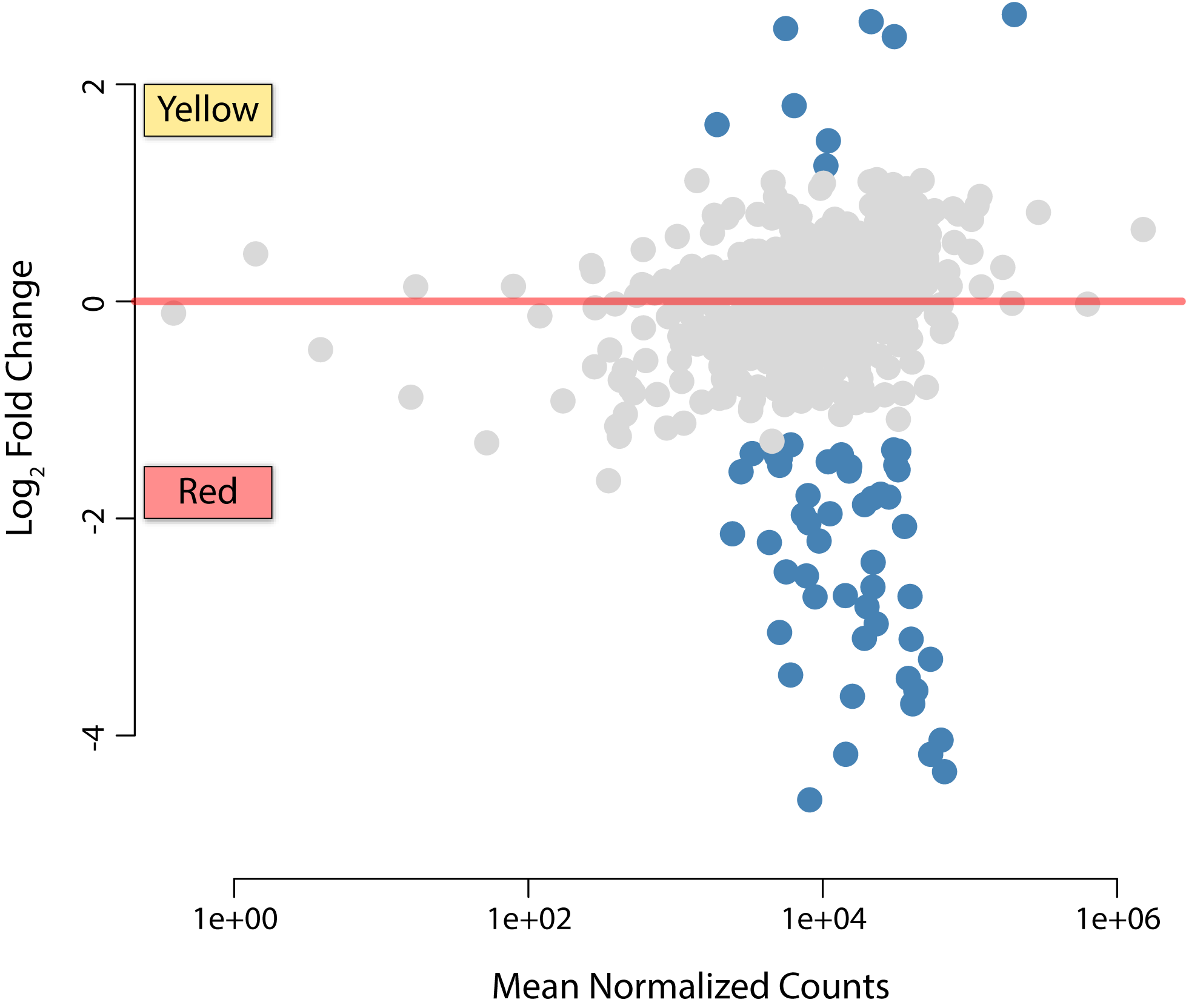

Supplement: Supplementary file 2 [file 3073FigureS2.tif]

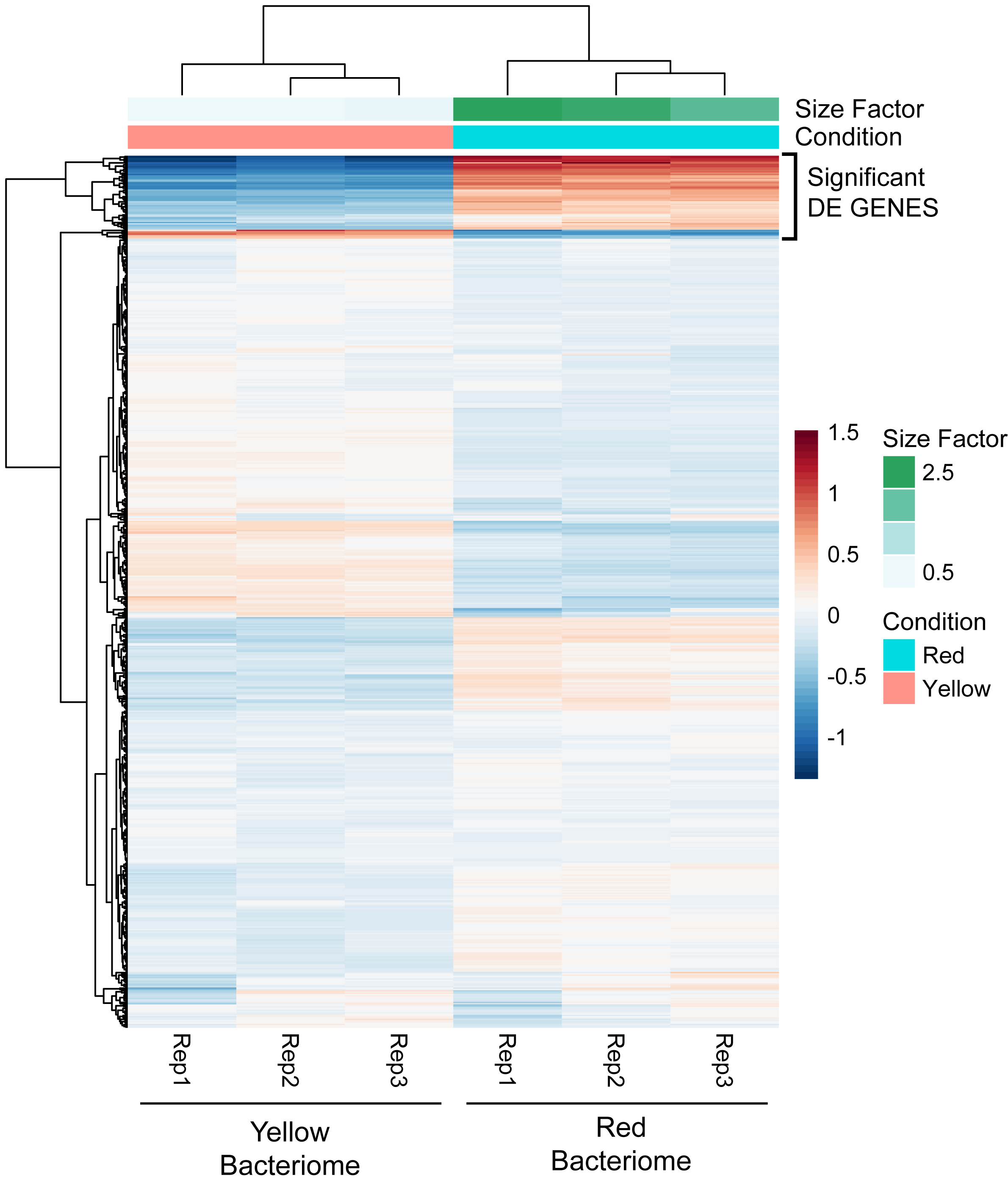

Supplement: Supplementary file 3 [file 3073FigureS3.tif]
